# NLRP3 inflammasome in neuroinflammation and central nervous system diseases

**DOI:** 10.1038/s41423-025-01275-w

**Published:** 2025-03-13

**Authors:** Wen Xu, Yi Huang, Rongbin Zhou

**Affiliations:** 1 Neurology Department, The First Affiliated Hospital of USTC, Division of Life Sciences and Medicine, University of Science and Technology of China, Hefei, Anhui 230001 P. R. China; 2 Institute of Health and Medicine, Hefei Comprehensive National Science Center, Hefei, 230601 China; 3 National Key Laboratory of Immune Response and Immunotherapy, Center for Advanced Interdisciplinary Science and Biomedicine of IHM, School of Basic Medical Sciences, Division of Life Sciences and Medicine, University of Science and Technology of China, Hefei, Anhui 230027 China; 4 Department of Geriatrics, Gerontology Institute of Anhui Province, The First Affiliated Hospital of University of Science and Technology of China, Division of Life Sciences and Medicine, University of Science and Technology of China, Hefei, Anhui 230001 China

**Keywords:** Neuroinflammation, NLRP3 inflammasome, Central nervous system (CNS) diseases, Neuroimmunology, Inflammasome

## Abstract

Neuroinflammation plays an important role in the pathogenesis of various central nervous system (CNS) diseases. The NLRP3 inflammasome is an important intracellular multiprotein complex composed of the innate immune receptor NLRP3, the adaptor protein ASC, and the protease caspase-1. The activation of the NLRP3 inflammasome can induce pyroptosis and the release of the proinflammatory cytokines IL-1β and IL-18, thus playing a central role in immune and inflammatory responses. Recent studies have revealed that the NLRP3 inflammasome is activated in the brain to induce neuroinflammation, leading to further neuronal damage and functional impairment, and contributes to the pathological process of various neurological diseases, such as multiple sclerosis, Parkinson’s disease, Alzheimer’s disease, and stroke. In this review, we summarize the important role of the NLRP3 inflammasome in the pathogenesis of neuroinflammation and the pathological course of CNS diseases and discuss potential approaches to target the NLRP3 inflammasome for the treatment of CNS diseases.

## Introduction

Neuroinflammation plays a crucial role in the pathogenesis of various central nervous system (CNS) diseases, which is not only a consequence of CNS disease progression but also one of the factors driving the progression of the disease [1]. NLRP3 (NOD-like receptor family pyrin domain containing 3) is a representative pattern recognition receptor that is widely expressed in microglia and astrocytes and forms an inflammasome to promote neuroinflammation upon sensing stress or damage. The NLRP3 inflammasome is an intracellular multiprotein complex whose activation in the brain mediates the inflammatory response that contributes to clearing damaged cells and promotes tissue repair [2, 3]. However, neuroinflammation induced by uncontrolled inflammasome activation also leads to neural dysfunction and even various CNS diseases, including multiple sclerosis, Parkinson’s disease, Alzheimer’s disease and stroke [4, 5]. Therefore, a deeper understanding of the mechanism by which the NLRP3 inflammasome is activated in the brain is critical for the development of therapeutic strategies for CNS diseases.

Currently, it is believed that the NLRP3 inflammasome can be activated through both the classical NLRP3 inflammasome activation pathway and nonclassical pathways [6–8]. The activation of the classical NLRP3 inflammasome requires two steps: the priming step and the activation step. In the priming step, Toll-like receptors (TLRs, such as TLR4) can induce the activation of the transcription factor NF-κB, promoting the expression of NLRP3 and pro-IL-1β. The activation step involves the recognition of NLRP3 inflammasome agonists, as well as the assembly and activation of the inflammasome. Upstream signals for NLRP3 activation and assembly include various molecules and cellular events, such as K^+^ efflux, Cl^−^ efflux, Na^+^ influx, mitochondrial dysfunction, the release of reactive oxygen species (ROS), mitochondrial DNA (mtDNA), and lysosomal damage [8]. The activation of the NLRP3 inflammasome begins with the recognition of pathogens or signals of cellular damage. When the body is invaded by pathogenic microorganisms or suffers from endogenous damage, NLRP3 is activated and subsequently recruits apoptosis-associated speck-like protein containing a CARD (ASC) and caspase-1 to form the inflammasome [9]. NEK7 (NIMA-related kinase 7) is a newly identified NLRP3 inflammasome component protein that promotes inflammasome assembly and caspase-1 activation by interacting with NLRP3 [10, 11]. Activated caspase-1 not only promotes the maturation of the inflammatory cytokines IL-1β and IL-18 but also promotes GSDMD cleavage, resulting in the translocation of its N-terminal domain to the cell membrane and the formation of pores to mediate pyroptosis, resulting in the release of the proinflammatory cytokines IL-1β and IL-18 [12–14]. Unlike the classical pathway, the nonclassical pathway relies on the activation of caspase-11 (caspase-4 and caspase-5 in humans). Under conditions of intracellular LPS stimulation, mouse caspase-11 and human caspase-4/5 can directly bind to the conserved structure of lipid A in LPS and become activated. Activated caspase-4/5/11 further cleaves the GSDMD protein, promoting pyroptosis. Interestingly, in the absence of Gasdermin D, activation of the NLRP3 inflammasome can trigger Gasdermin E, allowing the release of large amounts of IL-1β [15]. Caspase-3 can cleave GSDME to produce the N-terminal domain of Gasdermin E (GSDME-N), which has pore-forming activity, converting slow, noninflammatory apoptosis into rapid, inflammatory pyroptosis [16, 17]. Additionally, caspase-3 can be activated by caspase-8. Recent studies have shown that NLRP3 activation can initiate a proteolytic cascade involving the sequential cleavage of caspase-8, caspase-3, and GSDME [18].

Activation of the NLRP3 inflammasome in the brain is necessary for the clearance of invading pathogens and damaged cells and plays a neuroprotective role by promoting repair. However, when the NLRP3 inflammasome is abnormally activated or in an active state for a long period of time, it causes the continuous release of inflammatory factors to promote neuroinflammation. Chronic neuroinflammation can not only induce the death of nerve cells but also trigger the abnormal activation of glial cells, further promoting the expansion of the inflammatory response and thus damaging the function of the central nervous system [19, 20]. Therefore, understanding the role of the NLRP3 inflammasome in the occurrence of neuroinflammation and the pathological process of central nervous system diseases is important. On the one hand, exploring the activation mechanism of the NLRP3 inflammasome and how to effectively control its activation to prevent the exacerbation of neuroinflammation is highly important. On the other hand, the development of specific inhibitors or modulators targeting the NLRP3 inflammasome and its upstream and downstream pathways may become a new strategy for the treatment of central nervous system diseases.

## Neuroinflammation in central nervous system diseases

Neuroinflammation refers to the normal immune response generated within the CNS to harmful stimuli such as infection, injury, toxins, or autoimmunity, which is a major pathophysiological feature and key cause of many CNS diseases, including stroke, multiple sclerosis (MS), Alzheimer’s disease (AD), and Parkinson’s disease (PD) [21, 22]. Resident glial cells, including microglia, astrocytes, oligodendrocytes, and neurons, are involved in neuroinflammation [22–25]. Microglia are resident macrophages of the central nervous system that stabilize neural networks via the phagocytosis of neuronal debris and play crucial roles in synaptic remodeling and the maintenance of tissue homeostasis in a healthy brain [24, 26]. However, in the pathogenesis of central nervous system diseases, microglia can be activated by signals such as glutamate, high mobility box 1 (HMGB1) and nucleotides released by damaged neurons, inducing the production of proinflammatory cytokines, reactive oxygen species (ROS) and other substances that mediate neuroinflammation [24, 27–30]. Astrocytes are also important components of the innate immune system in the CNS. When the nervous system is injured, astrocytes typically undergo a process known as reactive astrogliosis, which mediates neuroinflammation. Moreover, they produce excessive inflammatory cytokines and reactive oxygen species, exacerbating neuronal cell death and disrupting neural circuits [23, 31–33].

Multiple sclerosis (MS) is an autoimmune disease of the central nervous system characterized by persistent inflammation and demyelination [34, 35]. In the early stages of MS, the continued activation of microglia leads to the production of proinflammatory cytokines, which in turn further induce microglial activation, thus exacerbating MS symptoms [36, 37]. LRP1 is expressed in the early pathological stage of MS, and the absence of LRP1 in microglia leads to increased secretion of the proinflammatory factor TNF-α, which exacerbates neuroinflammation [38]. Similarly, in the early and progressive stages of MS, activated microglia overexpress P2X7R, leading to the release of various proinflammatory cytokines, further exacerbating neuroinflammation [39]. In MS patients, the levels of colony-stimulating factor-1 receptor (CSF1R) and its ligand CSF1 are elevated in the CNS. CSF1R inhibitors significantly decrease the protein expression of IL-6 and IL-1β in microglia, thereby reducing neuroinflammation and alleviating MS [40]. In conclusion, these results suggest that the occurrence of neuroinflammation in the early stages of MS exacerbates the pathological process.

AD is another CNS disease that is closely associated with neuroinflammation, and various pathological factors, such as Aβ plaques, phosphorylated tau, proinflammatory cytokines, and oxidative stress, can activate microglia to induce neuroinflammation [41, 42]. Once activated by these pathological factors, microglia release many proinflammatory cytokines that trigger a strong inflammatory response. Continuously activated microglia may cause persistent inflammation, leading to neuronal damage and promoting the formation of amyloid beta (Aβ) plaques [43, 44]. Neuroinflammation is a significant pathological feature of PD and is characterized primarily by the activation of microglia in the CNS and the release of proinflammatory mediators Tables 1–3. This inflammatory cascade leads to the gradual loss of dopaminergic (DA) neurons, exacerbating motor dysfunction [45, 46].

**Table 1:** Targeting NLRP3 in central nervous system diseases

Therapeutic target	Inhibitor	Inhibitory concentration (IC_50_)	CNS diseases	Clinical trial phase	References
**ATPase activity of NLRP3**	CY-09	≈6 μM	Epilepsy, AD, Depress, CAPS, Stroke.	Preclinical	[112, 143–147]
	MCC950	7.5 nM	MS, AD, PD, Stroke, Spinal cord injury	Phase II	[72, 148–153]
	OLT1177 (Dapansutrile)	1 nM	MS, Intracerebral hemorrhage, AD, PD.	Phase II/III	[154–158]
	Tivantinib	1.2 μM	MS	Phase III	[159]
	BAY 11-7082	3 μM	AD, Traumatic brain injury	Phase I	[160, 161]
	Parthenolide	8.4 μM	MS, Traumatic brain injury	Phase II	[162–164]
**Interaction between NLRP3 and NEK7**	Oridonin	0.75 μM	Stroke, Epilepsy, AD, Depress.	Preclinical	[165–174]
	INF39	10 μM	Neuropathic pain	Preclinical	[175, 176]
**Oligomerization of NLRP3**	Tranilast	0.75 μM	Stroke, AD, CAPS, Traumatic brain injury	Phase II	[177–181]
	JC124	3.25 μM	Traumatic brain injury, AD	Preclinical	[182, 183]

**Table 2:** Targeting pyroptosis in central nervous system diseases

Therapeutic target	Inhibitor	CNS diseases	Clinical trial phase	References
**GSDMD**	Disulfiram	PD, MS, Glioblastoma	Phase II	[186–189]
	Dimethyl fumarate (DMF)	MS, Stroke	FDA approved	[190, 191]
	Necrosulfonamide (NSA)	Intracerebral hemorrhage, AD, PD.	Phase I	[192–195]
	Itaconate	CAPS	Preclinical	[196, 197]
	Phenethyl isothiocyanate (PEITC)	Glioblastoma	Phase II	[198, 199]
**GSDME**	Hypoxanthine	Stroke, AD	Preclinical	[202–204]

**Table 3:** Targeting ASC/NEK7 and other NLRP3 inflammasome components or effectors in CNS diseases

Therapeutic target	Inhibitors	CNS diseases	Clinical trial phase	References
**ASC**	Lycorine (LYC)	ALS,	Preclinical	[206, 207]
	Cardamonin	MS, AD	Preclinical	[208]
	Lonidamine (LND)	MS, Stroke	Preclinical	[209]
	*β*-Hydroxybutyrate (BHB)	AD	Preclinical	[210]
**NEK7**	Entrectinib	Gliomas	Preclinical	[214, 215]
	Berberine	MS, AD, PD, Stroke	Phase IV	[216–219]
	Licochalcone B	AD, Stroke	Preclinical	[220–222]
**Caspase-1**	VX-740 (Pralnacasan)/ VX-765	AD	Phase II	[104, 223, 224]
	Ac-YVAD-cmk	Intracerebral hemorrhage, Stroke	Preclinical	[225, 226]
	CZL80	Epilepsy	Preclinical	[227]
**IL-1β**	Canakinumab	CAPS	Phase III	[230, 231]

Stroke is a serious CNS disease with high incidence and mortality rates and can be classified into two main types: ischemic stroke and hemorrhagic stroke [47, 48]. Secondary injuries after a stroke include neuroinflammation, excitotoxicity, cell death, and disruption of the blood‒brain barrier (BBB) [49–52]. After a stroke, tissue damage releases many damage-related molecular patterns that lead to neuroinflammation in the damaged area of the brain, which increases oxidative stress, destroys the blood‒brain barrier, and directly leads to neuronal death [53]. Within minutes after a stroke, microglia are activated, undergo morphological changes, and secrete cytokines [54]. Moreover, astrocytes induce neuroinflammation by recruiting peripheral immune cells and releasing cytokines and chemokines [55, 56]. Additionally, leukocytes release proinflammatory cytokines, reactive oxygen species, and matrix metalloproteinases, further exacerbating neuroinflammation [57].

## NLRP3 inflammasome and neuroinflammation

Neuroinflammation, a key pathological cause of CNS diseases, has become a popular research topic in recent years. An increasing number of studies have shown that the NLRP3 inflammasome plays a crucial role in the pathogenesis of neuroinflammation [58].

**Fig. 1:**
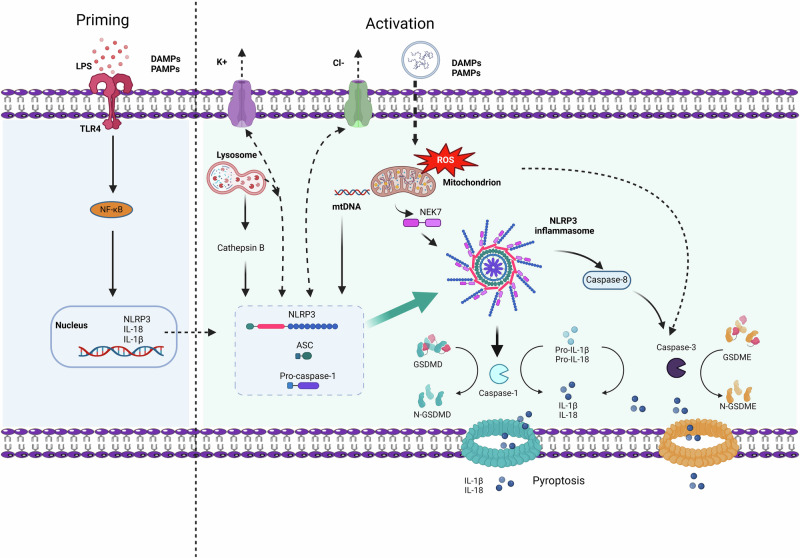
NLRP3 inflammasome activation pathway. Activation of the NLRP3 inflammasome requires two steps. The first step is priming (Left), triggered by LPS or DAMPs/PAMPs, which are recognized by TLR4 and induce the expression of interleukin 1β (IL-1β), IL-18, and NLRP3 through the activation of the transcription factor nuclear factor κB (NF-κB). The second step is the activation of the NLRP3 inflammasome (right), which is induced by various PAMPs and DAMPs and can activate multiple upstream signaling events. These include K^+^ efflux, Cl^-^ efflux, lysosomal rupture, mitochondrial dysfunction, the production of reactive oxygen species (mtROS), and the release of oxidized mitochondrial DNA (mtDNA). The activation of NLRP3 stimulates the assembly and oligomerization of the NLRP3 inflammasome complex, which further activates caspase-1, leading to the cleavage of pro-IL-1β and pro-IL-18. GSDMD is also cleaved, and N-GSDMD inserts into the membrane, forming pores and inducing pyroptosis while releasing inflammatory cytokines such as IL-1β and IL-18. Additionally, the activation of the NLRP3 inflammasome activates caspase-8 and caspase-3, cleaving GSDME, with the cleaved N-GSDME forming pores in the cell membrane and releasing IL-1β and IL-18. LPS lipopolysaccharide, PAMPs pathogen-associated molecular patterns, DAMPs damage-associated molecular patterns, TLR4 toll-like receptor 4, NF-κB nuclear factor-κB, ROS reactive oxygen species, mtDNA mitochondrial DNA, NLRP3 NOD-, LRR- and pyrin domain-containing protein 3, IL-1β interleukin 1β, IL-18 interleukin 18, NEK7 NIMA-related kinase 7, GSDMD gasdermin D, GSDME gasdermin E

NLRP3 is an important sensor in the innate immune system that not only recognizes the invasion of exogenous pathogens but also senses endogenous cell damage. NLRP3 is activated after experiencing upstream signals such as K^+^ and Cl^−^ efflux, mitochondrial dysfunction, the release of reactive ROS and mtDNA, and the release of cathepsin B. NLRP3 specifically interacts with NEK7, and after the activation of the inflammasome, the NEK7-NLRP3 interaction increases, leading to the oligomerization of NEK7 with NLRP3 into a complex. Oligomerized NLRP3 recruits ASC through PYD-PYD interactions and subsequently promotes the recruitment of procaspase-1 by ASC to form a protein complex called the NLRP3 inflammasome [9, 59]. After the NLRP3 inflammasome is activated, it induces the cleavage and activation of procaspase-1, leading to the maturation of the proinflammatory cytokines IL-1β and IL-18. Additionally, activated caspase-1 cleaves GSDMD and releases its N-terminal domain, which is translocated to the cell membrane, where it forms pores to induce pyroptosis and then mediates the release of cellular contents such as IL-1β and IL-18 (Fig. 1). These inflammatory factors not only induce local inflammatory responses but also may affect other organs through the bloodstream, further exacerbating systemic inflammation [8, 11, 12, 60].

Although activation of the NLRP3 inflammasome may play a protective role in the initial stages of neuroinflammation by clearing pathogens and cell debris, prolonged overactivation of the inflammasome can also lead to neurodegeneration [61, 62]. Chronic inflammation mediated by the NLRP3 inflammasome results in the sustained release of cytokines, leading to neuronal death and further compromising the homeostasis of the central nervous system (CNS). These findings highlight the complex role of NLRP3 in the neuroinflammatory process. In Alzheimer’s disease, the accumulation of amyloid β plaques triggers the activation of the NLRP3 inflammasome, resulting in increased production of IL-1β, which subsequently recruits immune cells and exacerbates neuronal damage [2, 63, 64]. Similarly, in Parkinson’s disease, the presence of α-synuclein aggregates is associated with the activation of NLRP3, underscoring the role of the inflammasome in the inflammatory environment surrounding pathological protein aggregation [65–67].

During neuroinflammation, activation of the NLRP3 inflammasome can lead to neuron damage and dysfunction. Excessive activation of NLRP3 can trigger an inflammatory response in glial cells, resulting in the release of many cytokines. These cytokines not only promote the progression of neuroinflammation but also may inhibit neuron survival and regeneration [68–70]. Furthermore, abnormal activation of NLRP3 is associated with pathological conditions such as stroke, trauma, and aging, where neuroinflammation further exacerbates disease progression [71–74]. Drugs targeting the NLRP3 inflammasome have shown promise in mouse models of CNS diseases. For example, the NLRP3 inhibitor MCC950 has demonstrated potential in improving inflammation and related pathology in neurodegenerative animal models [64, 75, 76]. These findings highlight the NLRP3 inflammasome as a viable therapeutic target with the potential to alleviate the impact of neuroinflammation in various CNS diseases.

In conclusion, the NLRP3 inflammasome plays a critical role in the pathogenesis of several CNS disorders by serving as a bridge between innate immunity and neuroinflammation. Understanding the dual function of the NLRP3 inflammasome as a preventative measure against acute inflammation and a cause of chronic neurodegeneration is crucial for the development of innovative treatment approaches. Importantly, the importance of the NLRP3 inflammasome in neuroinflammation should not be ignored, and more research is necessary to determine how its activation mechanisms are related to CNS disorders. To develop novel approaches and remedies for the prevention and management of neuroinflammation, future studies should aim to obtain a more thorough grasp of the activities and roles of NLRP3 in various neuropathological situations.

## NLRP3 inflammasome in central nervous system diseases

### NLRP3 in multiple sclerosis

Multiple sclerosis (MS) is a chronic autoimmune disorder characterized by progressive demyelination of the central nervous system, leading to severe neurological disabilities. According to a recent study, NLRP3 inflammasome-driven neuroinflammation contributes significantly to the pathogenic development of multiple sclerosis [77–79]. The peripheral caspase-1 mRNA level is associated with the severity of MS, and the level of caspase-1 is associated with the number of newly developed gadolinium-enhanced brain lesions [77]. MS patients also exhibit increased expression of caspase-1, IL-1β, and IL-18 [77]. Moreover, *Nlrp3* gene expression was significantly increased in a cuprizone-induced demyelination and neuroinflammation model, and defective Nlrp3 inhibited neuroinflammation and demyelination [78]. NLRP3 plays an important role in the induction of EAE, possibly through effects on caspase-1, subsequently influencing Th1 and Th17 cells [79]. Notably, RRMS patients had a much greater frequency of the NLRP3 rs3806265 C allele and CC genotype than did relapse patients and healthy controls, but remission patients had a significantly lower frequency, which demonstrated that these modifications in NLRP3-related molecular gene polymorphisms were linked to MS susceptibility [80].

**Fig. 2:**
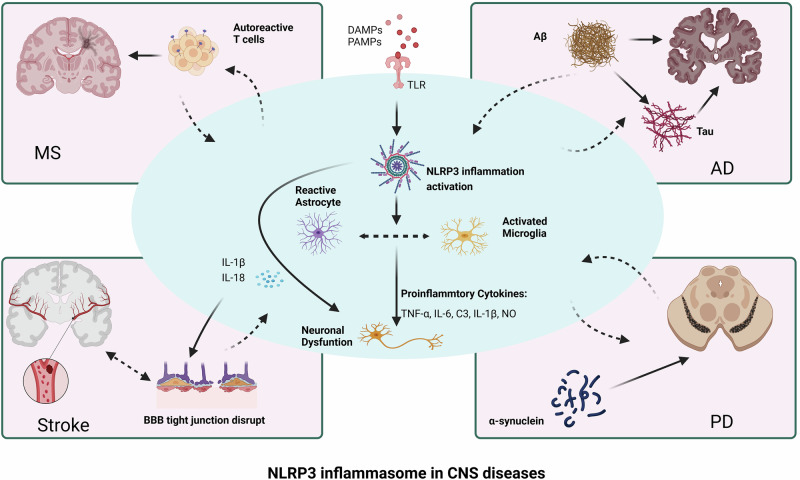
NLRP3 inflammasome in CNS diseases. The activation of the NLRP3 inflammasome in the CNS leads to the activation of microglia and astrocytes, and the inflammatory cytokines secreted by these cells further cause neuronal dysfunction. These outcomes exacerbate the infiltration of autoreactive T cells in the CNS during the pathogenesis of EAE, increase Aβ aggregation, induce tau pathology in AD, and cause α-synuclein aggregation in PD, leading to disruption of BBB tight junctions and a cascade of inflammatory responses in stroke. MS multiple sclerosis, AD Alzheimer’s disease, PD Parkinson’s disease, BBB blood‒brain barrier

In the pathogenesis of multiple sclerosis, the NLRP3 inflammasome plays a key role in inducing T-cell migration to the central nervous system. To acquire migratory ability, CD4(+) T cells need to be activated by sufficient antigen-presenting cells via the NLRP3 inflammasome, which in turn upregulates chemotactic-related proteins, and this process relies on NLRP3 [81]. Compared with wild-type mice, Nlrp3 knockout (KO) mice presented a decreased presence of Th1 and Th17 cells in both the spinal cord and peripheral lymphoid tissue, resulting in a significant reduction in symptoms associated with EAE [79, 81]. Zhang et al. reported that the specific loss of ASCs in microglia alleviated T-cell expansion and neutrophil recruitment during EAE pathogenesis [82]. Furthermore, peripheral blood monocyte RNA sequencing in 12 healthy controls and 44 untreated MS patients revealed that the NLRP3 inflammasome of monocytes in primary progressive MS patients was overactivated [83]. This activation then increases the production of IL-1β by binding lipopolysaccharide and ATP to the normal NLRP3 inflammasome. These findings suggest that the NLRP3 inflammasome may play an essential role in the development of EAE and serve as a prognostic marker for primary progressive EAE (Fig. 2).

NLRP3 inflammasome-independent IL-1β also plays a role in the progression of EAE. For example, IL-1β may influence EAE through the IL-1β-ARF6-ALK-SMAD1/5 pathway, which is independent of NLRP3 [84]. IL-17A can recruit myeloid cells that secrete IL-1β into the CNS, thereby inducing pathogenic γδT17 and Th17 cells and influencing the progression of EAE [85]. Deficiency of Pik3c3 in myeloid cells confers partial resistance to EAE, accompanied by a reduction in IL-1β production [86]. However, none of these targets have an impact on the progression of EAE as significantly as the NLRP3/IL-1β pathway does.

### NLRP3 in Parkinson’s disease

Parkinson’s disease (PD) is a rapidly increasing neurological disorder worldwide characterized by the progressive loss of dopaminergic (DA) neurons in the pars compacta nigra, the accumulation of α-synuclein in Lewy bodies and neurites, and excessive neuroinflammation [87]. The levels of the proinflammatory cytokines IL-1β and total α-synuclein in the plasma of PD patients are greater than those in the control group, and both are positively correlated with the severity of motor symptoms in PD patients [88]. These results demonstrate that α-synuclein and the inflammation-related cytokine IL-1β can serve as useful biomarkers for tracking the progression of Parkinson’s disease. Histological analysis of midbrain tissue from patients who died from PD revealed that the expression of NLRP3 and ASC was greater in degenerated midbrain tissue than in control tissue. Additionally, analysis of plasma from a multicenter cohort revealed that elevated levels of NLRP3 in plasma were associated with PD status [89]; furthermore, inflammasome-related proteins in plasma may serve as potentially useful biomarkers for patient stratification as well as for detecting and monitoring inflammation in PD patients. Although the above results suggest that NLRP3 inflammasome-derived neuroinflammation is involved in the onset and progression of PD, direct evidence was unclear until a study revealed that NLRP3 defects significantly inhibited neuroinflammation and the pathological processes of PD [3].

The components of the NLRP3 inflammasome are widely expressed in microglia, astrocytes, and neurons [90, 91]. In microglia, α-Syn fibrils and DA neuron degeneration work together to activate the NLRP3 inflammasome, and inhibition of NLRP3 with MCC950 dramatically decreases the quantity of α-Syn aggregates in the substantia nigra (SN) of mice, preventing the development of motor impairments and dopaminergic neuron degeneration in the SN [64]. There are also reports that NLRP3 within DA neurons is a substrate of Parkin, which typically inhibits the activation of the NLRP3 inflammasome by ubiquitinating the NLRP3 protein and promoting its proteasomal degradation [66]. The knockdown of caspase-1 can improve the loss of DA neurons and motor disorders in 1-methyl-4-phenyl-1,2,3,6-tetrahydropyridine/probenecid (MPTP/p)-induced PD model mice [92]. Furthermore, caspase-1 regulates DA neuron death through the caspase-7/PARP1/AIF pathway during the pathogenesis of PD in mice. These findings provide new insights into potential therapeutic targets for treating Parkinson’s disease via caspase-1 inhibition.

According to previous reports, the injection sides of PD rats given LPS and 6-OHDA presented elevated levels of NLRP3 inflammasome mRNA and protein expression, indicating that the NLRP3 inflammasome plays a role in the pathophysiology of 6-OHDA-induced PD in these mice [93]. By blocking downstream pathways such as the NLRP3/caspase-1/IL-1β axis, PD symptoms may occur less frequently, suggesting a novel avenue for prevention and treatment. In mouse models of PD, targeting NLRP3 effectively inhibits the occurrence and progression of parkin-induced PD in mice [65, 66]. The classic NLRP3 inhibitor MCC950 has been confirmed to have a protective effect in PD models. Other small molecule inhibitors, such as kaempferol (Ka), astragaloside IV, and andrographolide, have also been shown to inhibit the progression of PD in animal models [67, 94, 95]. However, their safety and efficacy in clinical settings remain to be explored.

### NLRP3 in Alzheimer’s disease

Alzheimer’s disease (AD) is a major progressive neurodegenerative disorder characterized by the presence of extracellular amyloid beta (Aβ) plaques and intracellular hyperphosphorylated tau protein tangles in the brain. The main clinical manifestations are progressive dementia and other cognitive impairments. In recent years, the number of AD patients has increased rapidly, with more than 50 million people living with dementia worldwide, and this number is expected to increase to 152 million by 2050, seriously endangering life and health [96–98]. Therefore, an in-depth study of the pathological mechanism of AD is essential for the development of intervention strategies.

NLRP3 inflammasome-driven neuroinflammation has recently been reported to contribute to the pathological process of AD. Blocking NLRP3 in tau transgenic mice can reduce the excessive phosphorylation and aggregation of tau by affecting the activity of certain tau kinases and phosphatases [99]. Additionally, inhibiting NLRP3 can promote pericyte survival, improve cerebrovascular function, and alleviate AD pathology in the brains of tau transgenic mice [100]. These results highlight the significant role of NLRP3 inflammasome activation in the pathogenesis of tauopathies. Compared with that in healthy controls, the expression of IL-1β and GSDMD in the cerebrospinal fluid (CSF) of AD patients is significantly increased [101]. Similar results were also reported in the brain tissue and peripheral blood mononuclear cells of AD patients [102–104]. Studies have shown that oligodendroglial glycolytic stress induces inflammasome activation during the pathological process of AD [105, 106]. Aβ triggers caspase B release, which in turn triggers the NLRP3 inflammasome and promotes the release of IL-1β and IL-18. Furthermore, blocking NLRP3 can ameliorate memory loss and reduce Aβ deposition. In the APP/PS1 animal model [107], researchers discovered a direct correlation between the activation of the NLRP3 inflammasome and initial Aβ deposition at six months, confirming the strong association between NLRP3 and Aβ. Furthermore, Aβ deposition in AD patients is linked to innate immune system activation and plays a role in the development of inflammasome-dependent ASCs in microglia [108]. While the use of anti-ASC antibodies stops Aβ accumulation and AD progression, the ASC spots released by microglia quickly bind to Aβ, increasing the formation of Aβ oligomers and aggregates.

Recent studies have confirmed that multiple small-molecule drugs targeting the NLRP3 pathway can reduce Aβ deposition and improve AD disease progression [75, 109, 110]. MCC950 effectively blocks the activation of the NLRP3 inflammasome induced by Aβ or Tau, preventing the cleavage and release of caspase-1 and IL-1β, reducing Aβ deposition, and improving memory deficits and other symptoms in the APP/PS1 model of AD. Anakinra (a recombinant IL-1 receptor antagonist) can reduce Aβ and Tau deposition and lower IL-1β, thereby alleviating cognitive deficits in the 3xTg AD model [111]. Blocking IL-1β or interleukin receptor-1 knockout (Il1r1^-/-^) can also alleviate Aβ-induced mitochondrial dysfunction and memory impairment both in vivo and in vitro, respectively [112]. In addition, the caspase-1 inhibitor VX-765 rescued spatial memory deficits in mice aged 5–8 months, reduced glial proliferation, and significantly diminished Aβ deposition [113]. When VX-765 treatment was stopped, memory deficits recurred in older AD model mice (12–15 months). VX-765 can also significantly improve the cognitive and spatial memory deficits observed in aged AD model mice, which exhibit substantial Aβ accumulation and microglial inflammation [114]. Together, these results suggest that the NLRP3 inflammasome is a potential intervention target for AD.

### NLRP3 in stroke

Stroke is a common CNS disease and one of the leading causes of disability and death worldwide. Ischemic stroke accounts for 60–70% of all strokes [115, 116], yet it is the second leading cause of death. This study examined brain tissue samples from stroke patients and revealed increased levels of IL-1β, IL-18, and the NLRP3 inflammasome protein. Similarly, in an animal model of stroke, Franke et al reported that the expression of NLRP3 and other NLRP3 inflammasome-related genes increased 20–30-fold within 24 h after stroke in 2021 [117]. Intravenous immunoglobulin (IVIg) treatment was found to protect neurons in these models by inhibiting the activity of the NLRP1 and the NLRP3 inflammasome [118]. Focal ischemia was induced in NLRP3^-/-^ or WT mice through middle cerebral artery occlusion [119]. The absence of NLRP3 can ameliorate brain injury in mice after ischemic stroke by reducing infarction and blood‒brain barrier (BBB) damage.

The activation of the NLRP3 inflammasome is a core stage of neuroinflammation and pyroptosis and a key process associated with poststroke brain injury. The extensive involvement of the NLRP3 inflammasome in brain damage following stroke makes it a significant target for treatment. By blocking NLRP3 activation, MCC950 can improve stroke outcomes in a mouse model of transient middle cerebral artery occlusion (tMCAO) [120]. Treatment with MCC950 after stroke can reduce the expression of various proinflammatory cytokines and components of the NLRP3 inflammasome, thereby decreasing infarct volume in a dose-dependent manner [121]. Another typical NLRP3 inflammasome inhibitor, CY-09, was also found to improve brain inflammatory cell death after stroke [122]. Moreover, inhibiting Bruton’s tyrosine kinase (BTK), an upstream activator of the NLRP3 inflammasome, can reduce the infarct volume and associated neurological damage following ischemic stroke [123]. In addition, a series of natural chemical substances play important roles in models of stroke by inhibiting NLRP3 inflammasome signaling [124–126]. Curcumin, sinomenine and chrysophanol can alleviate brain damage and neurological deficits following ischemic stroke while also reducing the formation of the NLRP3 inflammasome and related signaling pathways [124–126].

### NLRP3 in other central nervous system diseases

The NLRP3 inflammasome is also involved in the onset and progression of other CNS diseases, such as depression, autoimmune encephalitis, and epilepsy [127–129].

Substantial evidence indicates that the NLRP3 inflammasome is activated in the brain and blood samples of patients with depression [130, 131]. Stress-induced depression triggers the activation of the NLRP3 inflammasome in the hippocampus to increase the expression of IL-1β [132, 133]. Depression models induced by other methods in mice also exhibit neuroinflammation related to the NLRP3 inflammasome in the hippocampus [134–136], suggesting that the serum NLRP3 inflammasome can be used to detect and assess the severity of depression [137]. In the absence of NLRP3, chronic stress does not cause depressive behaviors or microglial activation in mice, nor does it inhibit hippocampal neurogenesis [138]. Many compounds, such as fluoxetine, MCC950 and berberine, block depression by inhibiting the NLRP3 inflammasome [139]. Fluoxetine exerts its therapeutic effects by targeting the antioxidant Nrf2/HO-1 and inhibiting the TLR4/NLRP3 inflammasome signaling pathway [140]. Both NLRP3 knockout and MCC950 significantly improved the extinction of contextual fear memory and reduced anxiety-like behaviors [141]. Berberine improves depression-like behavior in mice by inhibiting the activation of the NLRP3 inflammasome and rescues neuronal damage by preventing synaptic plasticity and neurogenesis impairments [142].

Anti-N-methyl-D-aspartate receptor (NMDAR) encephalitis is the most common autoimmune encephalitis. Compared with control patients, patients with anti-NMDAR encephalitis presented significantly greater serum levels of NLRP3, IL-1β, and IL-18. IL-18 and NLRP3 in serum are positively associated with the mRS score during the acute phase of encephalitis, suggesting that the pathophysiology of NMDAR encephalitis may be significantly influenced by the NLRP3 inflammasome pathway [143]. Overactivation of the NLRP3 signaling pathway was found to promote the inflammatory response and cognitive dysfunction in mice with NMDAR encephalitis [144]. Correspondingly, blocking the NLRP3 inflammasome through MCC950 has a potential protective effect against NMDAR encephalitis [145], suggesting that the NLRP3 inflammasome may be an important indicator for determining disease prognosis.

Numerous preclinical studies have confirmed that the NLRP3 inflammasome pathway is closely related to the development of epilepsy [129, 146]. GPR120 regulates neuroinflammation through the NLRP3/Caspase-1/IL-1β signaling pathway in an epilepsy animal model [146]. Seizure stimulation can lead to the expression of the NLRP3 inflammasome in neurons and induce downstream neuroinflammation. Pharmacological or genetic blockade of NLRP3 may improve the progression of epilepsy by alleviating neuroinflammation [147]. Rapamycin can improve the seizure threshold and latency by modulating the NLRP3 inflammasome, thereby alleviating seizure symptoms [148]. Combined treatment with valproic acid and furosemide significantly reduces the protein expression levels of ASC and NLRP3 and decreases the severity of epilepsy in rats [149].

## Treatments targeting NLRP3 in central nervous system diseases

### Targeting NLRP3

As an important innate immune receptor, NLRP3 is the core component of the NLRP3 inflammasome. After specific stimulus signals are sensed, NLRP3 promotes interaction with ASC, thereby recruiting and activating caspase-1 and inducing the formation of the inflammasome complex [150, 151]. NLRP3 inflammasome-mediated neuroinflammation contributes to the pathological progression of CNS diseases, making NLRP3 an attractive therapeutic target for CNS diseases.

#### Inhibition of the ATPase activity of NLRP3

CY-09, an analog of the cystic fibrosis transmembrane conductance regulator (CFTR) channel inhibitor CFTRinh-172, is the first small-molecule compound reported to directly bind NLRP3 to inhibit inflammasome activation and has anti-inflammatory activity in vivo [152, 153]. In 2017, Jiang reported that CY-09 directly binds to the ATP-binding motif of the NACHT domain in NLRP3 and inhibits its ATPase activity, thereby suppressing the assembly and activation of the NLRP3 inflammasome [153]. As the first NLRP3 inflammasome-targeted inhibitor, CY-09 is widely used to treat CNS diseases because it inhibits neuroinflammation [153]. CY-09 reportedly improves pentylenetetrazol-induced epilepsy and neuronal loss in mice by attenuating NLRP3-dependent neuroinflammation, suggesting that CY-09 may be a promising drug for the treatment of epilepsy [154]. CY-09 helps improve pathology and alleviate cognitive deficits in 3×Tg-AD mice [155]. Moreover, CY-09 also exerts antidepressant-like effects by reducing neuroinflammation in microglia [156]. In addition, CY-09 regulates NLRP3-mediated neuronal pyroptosis after cerebral ischemia/reperfusion injury by affecting the low-density lipoprotein receptor (LDLR) [122].

MCC950 is a representative and highly effective NLRP3 inhibitor that suppresses NLRP3 activation at nanomolar concentrations [76]. Mechanistically, MCC950 was confirmed to inhibit ATP hydrolysis by directly interacting with the Walker B motif of the NACHT domain in NLRP3, thereby inhibiting the activation of the NLRP3 inflammasome [157, 158]. MCC950 has good therapeutic effects on CNS diseases in animals. MCC950 can alleviate IL-1β signaling and T-cell-mediated inflammatory responses in EAE by directly inhibiting NLRP3 activation [159]. In addition, MCC950 exerts neuroprotective effects by inhibiting the activation of neuronal the NLRP3 inflammasome, reducing neuronal apoptosis, alleviating the severity of damage, and promoting the recovery of motor function after SCI [160]. Similar protective effects have been demonstrated in multiple animal models of Parkinson’s disease to alleviate motor dysfunction, nigrostriatal dopaminergic degeneration, and the accumulation of α-synuclein aggregates [64]. In addition to its effects on CNS diseases described above, MCC950 also has a good remission effect on the pathological process of AD [161], improves neurovascular remodeling and prevents the worsening of cognitive decline after stroke [162].

OLT1177 (dapansutrile) is an orally active β-sulfonyl nitrile compound that prevents the interaction between NLRP3 and ASC, as well as between NLRP3 and caspase-1, by reducing the ATPase activity of NLRP3 [163]. Oral OLT1177 significantly improves the clinical symptoms of EAE by reducing the infiltration of CD4^+^ T cells and macrophages in the spinal cord [164]. A mechanistic study revealed that OLT1177 significantly reduced the levels of IL-1β and IL-18 in the spinal cords of EAE mice by inhibiting the activation of NLRP3. OLT1177 significantly alleviated brain edema after intracerebral hemorrhage in mice, maintained the integrity of the blood‒brain barrier, and reduced vascular leakage [165]. Moreover, OLT1177 preserved the morphological transformation of microglia and significantly inhibited the activation of caspase-1 and the release of IL-1β by inhibiting NLRP3. OLT1177 can improve neuroinflammation by lowering microglial activity, reducing the number of plaques in the cortex, and restoring synaptic plasticity in an AD animal model [166]. In the MPTP model of PD, treatment with OLT1177 can prevent the loss of motor function by inhibiting NLRP3, reducing α-synuclein levels, modulating proinflammatory markers in the substantia nigra-striatum region, and protecting dopaminergic neurons from degeneration, thereby improving PD symptoms [167]. We found that the anticancer drug tivantinib inhibits the NLRP3 inflammasome by directly blocking NLRP3 ATPase activity and subsequent inflammasome complex assembly. In vivo, tivantinib also has significant preventive and therapeutic effects on EAE. As a specific inhibitor of NLRP3 [168], tivantinib is a promising therapeutic agent for inflammasome-driven diseases. BAY 11--7082 inhibits NLRP3 activation by downregulating NLRP3 ATPase activity [169]. Treatment with BAY 11-7082 significantly reduced caspase-1 and IL-1β levels in a traumatic brain injury mouse model. Furthermore, in an Alzheimer’s disease mouse model [170], BAY 11--7082 also inhibited NLRP3 inflammasome activation, thereby alleviating neuronal damage and cognitive dysfunction. Parthenolide is a plant sesquiterpene lactone with various anti-inflammatory properties that inhibits the activation of multiple inflammasomes in macrophages by inhibiting the ATPase activity of NLRP3 [171]. By inhibiting the activation of inflammasomes, parthenolide improves neurological deficits and neuroinflammation in traumatic brain injury model mice [172]. Moreover, it can inhibit Th 17 cells and alleviate EAE [173].

#### Blocking the interaction between NLRP3 and NEK7

Oridonin is the main bioactive component of the herbal plant Rabdosia Rubescens [174]. It is a bioactive ent-kaurane diterpenoid compound that has antitumor, anti-inflammatory, and proapoptotic effects [175–177]. Our preliminary research revealed that oridonin forms a covalent bond with cysteine 279 in the NACHT domain of NLRP3, blocking the interaction between NLRP3 and NEK7 and thereby inhibiting the assembly and activation of the NLRP3 inflammasome [178]. Moreover, in vivo experiments revealed that oridonin has preventive or therapeutic effects on mouse peritonitis, gouty arthritis, and type 2 diabetes models through the inhibition of NLRP3 activation. In addition to the aforementioned inflammatory diseases, oridonin has also been shown to protect against a variety of CNS diseases by inhibiting the NLRP3 inflammasome. Oridonin was found to rescue early neuronal loss in ischemic stroke [179] and alleviate synaptic loss and memory deficits in AD model mice induced by Aβ1‒42 [180, 181]. Oridonin exerts antidepressant effects by regulating PPAR-γ/AMPA receptor signaling through the inhibition of NLRP3 [182]. Furthermore, oridonin has a neuroprotective effect by blocking pyroptosis in a mouse model of chronic epilepsy via the NLRP3/caspase-1 pathway [183].

INF39 is a nontoxic, irreversible acrylic ester-induced NLRP3 inflammasome inhibitor that specifically inhibits NLRP3 activation without affecting the NLRC4 or AIM2 inflammasome [184]. INF39 does not have a direct inhibitory effect on events upstream or downstream of the inflammasome [184]. Instead, it inhibits inflammasome assembly by blocking the interaction between NEK7 and NLRP3. The NLRP3 inhibitor INF39 can inhibit the function of the recombinant protein CXCL13 in primary rat astrocytes and ameliorate neuropathic pain in rats [185].

#### Preventing the oligomerization of NLRP3

Tranilast is an analog of tryptophan metabolites and was initially identified as an antihistamine [186]. It is used to treat inflammatory diseases such as bronchial asthma, atypical dermatitis, and allergic conjunctivitis. Our previous study revealed that tranilast directly binds to the NACHT domain of NLRP3, inhibiting inflammasome assembly and activation by blocking NLRP3 oligomerization [187]. It has been reported that tranilast improves the prognosis of ischemic stroke patients by stimulating M2 polarization of microglia, improving sensorimotor function and inhibiting the production of inflammatory cytokines, thereby reducing infarct size in stroke model mice [188]. Tranilast can alleviate the cognitive impairment induced by Aβ [189]. A rolipram-tranilast hybrid significantly increases the levels of heme oxygenase-1 (HO-1) while downregulating the expression of phosphodiesterase-4B (PDE4B) [190]. It markedly reduces traumatic brain injury and has good neuroprotective effects.

JC124 is a selective NLRP3 inflammasome inhibitor that directly targets the NLRP3 inflammasome complex without altering its ATPase activity [191]. Treatment with JC124 dramatically decreases the extent of cortical lesions, the inflammatory cell response in the wounded brain, and the number of degenerative neurons induced by traumatic brain injury [191]. Rats treated with JC-124 also presented significant decreases in the protein expression levels of NLRP3, ASC, IL-1β, and caspase-1 in their brain tissue. The soluble and insoluble Aβ 1-42 levels in the brains of AD model mice, as well as the amount of Aβ deposition, can be decreased by JC-124 therapy. In addition, astrocyte proliferation is increased, whereas APP β cleavage and microglial activation are decreased [192]. This finding illustrates how JC-124, a particular NLRP3 inflammasome inhibitor, protects the AD mouse model.

### Targeting pyroptosis: GSDMD/GSDME

#### Targeting GSDMD

GSDMD is a key executor of pyroptosis, which can be cleaved by caspase-1 activated by the inflammasome, and the released N-terminal domain is transferred to the cell membrane to form pores, inducing pyroptosis and mediating the release of the proinflammatory cytokines IL-1β and IL-18 [193]. The primary function of pyroptosis is to protect the host from microbial infections. However, excessive pyroptosis can lead to various inflammatory diseases, including CNS disorders. Therefore, treatments targeting GSDMD are important for treating CNS diseases [194]. For example, drugs such as disulfiram [195], dimethyl fumarate (DMF) [196], necrosulfonamide (NSA) [197], itaconate [198], and phenethyl isothiocyanate (PEITC) can target GSDMD to treat CNS diseases [199].

Disulfiram (a medication for treating alcohol addiction) is an inhibitor of GSDMD pore formation but not other members of the GSDM family [195, 200]. Disulfiram covalently modifies human/mouse Cys191/Cys192 in GSDMD to block pore formation, thereby preventing IL-1β release and pyroptosis. Disulfiram effectively inhibits dopaminergic neuron death and alleviates microglial activation, which mitigates the pathology of PD [201]. Moreover, disulfiram can inhibit the progression of EAE by downregulating Th17 levels [202].

Dimethyl fumarate (DMF) has long been used in the clinical treatment of multiple sclerosis, but the mechanism is not fully understood. Recent research has shown that dimethyl fumarate (DMF) or endogenous fumarate reacts with GSDMD at a critical cysteine residue to form S-(2-succinyl)-cysteine [196]. The subsequent succinylated GSDMD prevents its interaction with caspases, thus limiting its ability to induce pyroptosis. DMF can alleviate familial Mediterranean fever and experimental autoimmune encephalitis by targeting GSDMD. In addition, DMF can alter the immune environment in the peripheral and central nervous systems after ischemic stroke, reduce the volume of acute infarction, promote the recruitment of microglia, and protect neurons [203].

Necrosulfonamide (NSA) has recently been found to bind to GSDMD at Cys191, thereby preventing macrophage pyroptosis by inhibiting the oligomerization of GSDMD [204]. In addition, pretreatment with NSA can suppress the pyroptosis of cortical neurons induced by _Aβ1‒42_, and this inhibitory effect mainly involves the targeting of GSDMD and the release of inflammatory factors [197]. NSA also inhibited the oligomerization and phosphorylation of α-synuclein in the substantia nigra of PD mice by suppressing the activation of microglia and reactive astrogliosis [205]. NSA has a neuroprotective effect on alleviating acute brain injury in a mouse model of cerebral hemorrhage by reducing neuroinflammation [206].

In addition to the above compounds, several other molecules have been reported to inhibit pyroptosis by targeting GSDMD. Itaconate is an intracellular metabolite with a Michael acceptor structure. Artyomov et al. reported that endogenous itaconate directly binds to GSDMD by covalently modifying the thiol group of Cys77 and may interfere with the caspase-GSDMD interaction, thereby inhibiting pyroptosis and tissue damage [207]. Itaconate inhibited the release of NLRP3-dependent IL-1β from PBMCs isolated from patients with cold-induced autoinflammatory syndrome (CAPS) [198]. Phenethyl isothiocyanate (PEITC) is a metabolite found in cruciferous vegetables and has been identified as a covalent inhibitor of GSDMD. PEITC can reduce the production of NLRP3 as well as the cleavage of caspase-1 and GSDMD in a mouse model of acute liver injury and in vitro [199]. Moreover, phenethyl isothiocyanate (PEITC) inhibits the migration and invasion of human glioblastoma GBM 8401 cells [208].

#### Targeting GSDME

Gasdermin E (GSDME) is another important member of the gasdermin protein family, which also mediates pyroptosis, similar to GSDMD [209]. GSDME is specifically cleaved by caspase-3 at its linker region to produce the GSDME-N fragment, which permeates the plasma membrane and converts slow, noninflammatory apoptosis into rapid, inflammatory pyroptosis [210]. GSDME-mediated pyroptosis is closely associated with CNS diseases, and targeting GSDME may provide potential therapeutic strategies for these diseases. Hypoxanthine, an important metabolite of purine metabolism, has been reported to cause vascular damage and disruption of the blood‒brain barrier (BBB) in stroke by regulating intracellular Ca^2+^ overload and inducing GSDME-dependent endothelial cell pyroptosis [211]. Maresin1 alleviates cerebral ischemia‒reperfusion injury by inhibiting caspase-3/GSDME-mediated pyroptosis and neuroinflammation [212]. Aβ treatment induces pyroptosis in SH-SY5Y cells through active caspase-3/GSDME. GSDME knockdown improved cognitive impairment in APP23/PS45 mice by alleviating the inflammatory response [213].

Currently, research on GSDME inhibitors faces many challenges. The development and use of inhibitors are influenced by two factors: the molecular mechanisms of inhibition targeting GSDME are not yet fully understood. Additionally, blocking GSDME may affect the physiological functions of healthy cells, which could lead to negative consequences. We anticipate further research on the impact of GSDME inhibitors on central nervous system diseases.

### Targeting other components: ASC, NEK7, caspase-1 and IL-1β

#### Targeting ASCs

ASC is an important component of the inflammasome and can act as an adaptor protein to recruit NLRP3 and caspase-1 to promote inflammasome assembly and activation [214]. ASC has been widely reported to be involved in a variety of CNS diseases. Microglia-released ASCs can rapidly bind to Aβ, increasing the formation of Aβ oligomers and aggregates. Blocking ASCs with antibodies can prevent the increase in Aβ pathology in AD mice, suggesting that targeting ASCs may be an important strategy for treating CNS diseases [107]. Lycorine (LYC), cardamonin, lonidamine (LND), and β-hydroxybutyrate (BHB) are inhibitors that target ASC.

Lycorine (LYC) is an alkaloid isolated from plants of the Amaryllidaceae family that is known for its effective anti-inflammatory activity. Recent mechanistic studies have shown that LYC improves bleomycin-induced pulmonary fibrosis by inhibiting NLRP3 inflammasome activation and pyroptosis through targeting the PYD domain of ASC [215]. Lycorine is a promising lead compound for the treatment of ALS because it inhibits the synthesis of TDP-43 A315T [216]. Similarly, Cardamonin, the active ingredient of the traditional Chinese medicinal herb Alpinia galanga, was found to specifically block ASC oligomerization and the NLRP3 inflammasome and has a good effect on NLRP3 inflammasome-driven disease [217]. In addition, the antitumor drug lonidamine (LND) directly binds to ASC and inhibits its oligomerization, thereby suppressing NLRP3 activation [218]. In vivo experiments have shown that LND significantly reduces inflammatory damage in experimental models of inflammasome-related diseases, including EAE, ischemic stroke, and sepsis [218]. β-Hydroxybutyrate (BHB) inhibits NLRP3 inflammasome activity by reducing the assembly of ASC oligomers and the formation of specks. The administration of BHB to 5xFAD mice can decrease the formation of Aβ plaques, microglial proliferation, and caspase-1 activation [219].

#### Targeting NEK7

NEK7 is an essential mediator of NLRP3 inflammasome activation downstream of potassium efflux, which initiates inflammasome assembly and activation by interacting with NLRP3 [10, 220]. Therefore, inhibitors targeting NEK7 are important for suppressing the activation of the inflammasome and related CNS diseases [221, 222]. It has been reported that entrectinib, berberine, and licochalcone B inhibit the activation of the inflammasome by targeting NEK7.

Entrectinib is a US Food and Drug Administration (FDA)-approved anticancer agent, and our recent study revealed that it inhibits NLRP3 inflammasome activation [223]. A mechanistic study revealed that entrectinib directly binds to the arginine 121 residue (R121) of NEK7, blocking the interaction between NEK7 and NLRP3 and thereby inhibiting the assembly and activation of the inflammasome. In vivo studies also indicate that entrectinib significantly improves animal models of NLRP3 inflammasome-related diseases, such as lipopolysaccharide-induced systemic inflammation, peritonitis, and type 2 diabetes [223]. Entrectinib has been approved for clinical use in the treatment of high-grade gliomas [224]. Berberine, a traditional Chinese medicine, has therapeutic effects on various inflammation-related diseases, but its mechanism of action is unknown. A recent study revealed that berberine directly targets the NEK7 protein, specifically by blocking the NEK7-NLRP3 interaction and continuously inhibiting IL-1β release. Furthermore, berberine exhibited anti-inflammatory effects in vivo in a NEK7-dependent manner. These findings suggest that NEK7 is a key target for berberine in the treatment of NLRP3-related inflammatory diseases [225]. Berberine effectively improves EAE and alleviates neuroinflammation [226], and it was also confirmed that it significantly improved symptoms in AD and PD models [227, 228]. Licochalcone B can also directly bind to NEK7 and inhibit the interaction between NLRP3 and NEK7, exerting anti-inflammatory effects [229]. It also effectively improves symptoms of Parkinson’s disease and stroke while alleviating inflammation [230, 231].

#### Targeting caspase-1

Caspase-1 is an important cysteine protease that plays a key role in activating inflammasomes and processing proinflammatory cytokines. Recently, inhibitors targeting caspase-1 have shown good efficacy in various inflammation-related diseases. These include VX-740 (Pralnacasan)/VX-765, Ac-YVAD-cmk and CZL80.

VX-740 (Pralnacasan) and its analog VX-765 are inhibitors of caspase-1. Both act by covalently modifying the catalytic cysteine residue at the active site of caspase-1, thereby blocking caspase-1 cleavage and the resulting pro-IL-1β/18 [232, 233]. It also showed good anti-inflammatory effects and therapeutic results in mouse models for the treatment of rheumatoid arthritis (RA) and osteoarthritis (OA) [232]. The small molecule caspase-1 inhibitor VX-765 reversed the deficits in contextual and spatial memory in the AD J20 mouse model in a dose-dependent manner and ameliorated the effects of neuroinflammation and Aβ accumulation. These results provide evidence for the efficacy of Caspase-1 inhibition against cognitive deficits and pathology in Alzheimer’s disease [113]. Ac-YVAD-CMk, a selective irreversible inhibitor of caspase-1, can inhibit the activation of procaspase-1 after cerebral hemorrhage, reduce cerebral edema, decrease the activation level of microglia, lower the expression of inflammation-related factors, and improve the behavioral performance of rats with cerebral hemorrhage [234]. Ac-YVAD-CMK significantly improved cognitive function in mice after stroke and reversed changes in hippocampal brain volume by inhibiting caspase-1 [235]. Another important caspase-1 inhibitor, CZL80, terminates diazepam-resistant status epilepticus by blocking glutamatergic transmission, which is highly important for the clinical treatment of refractory status epilepticus [236].

#### Targeting IL-1β

IL-1β is a proinflammatory cytokine that mediates the inflammatory response after the activation of the inflammasome, and excessive production of IL-1β can cause a variety of CNS diseases [237]. Drugs that block IL-1β signaling have been shown to be beneficial in many neuroinflammatory diseases, making IL-1β a promising therapeutic target for these diseases [238]. Canakinumab is a human anti-IL-1β monoclonal antibody that inhibits inflammation in patients with autoimmune diseases by neutralizing IL-1β signaling [239]. This herb is used to treat cryopyrin-associated periodic syndrome (clinical features include urticarial rash and fever, with the central nervous system and musculoskeletal system) [240].

## Conclusions and future perspective

In conclusion, the NLRP3 inflammasome plays a complex and crucial role in the pathophysiology of diseases of the central nervous system, including multiple sclerosis, Alzheimer’s disease, Parkinson’s disease, and stroke. Its formation and activation are accompanied by the production of IL-1β and IL-18, as well as pyroptosis, to mediate the inflammatory response. In addition to playing a significant role in the development of neurological disorders, it may also be a target for treatment. In-depth research into the mechanisms of the NLRP3 inflammasome and its role in various CNS diseases will help us better understand the nature of neuroinflammation. Moreover, inhibitors targeting NLRP3 and other components involved in the activation of the NLRP3 inflammasome (such as GSDMD, GSDME, NEK7, ASC, caspase-1 and IL-1β) are continuously being developed, providing new ideas and strategies for the prevention and treatment of central nervous system diseases.

Although many compounds have been reported to alleviate CNS disease by inhibiting the NLRP3 inflammasome, several key challenges remain. First, ASC, caspase-1, GSDMD and IL-1β are common components of various inflammasomes, and the development of NLRP3 inflammasome inhibitory drugs that target these molecules for the treatment of CNS diseases may have off-target effects. Second, the blood‒brain barrier protects the brain from damage, but it also inevitably blocks drugs from entering the brain to play a role. How small-molecule drugs can alleviate CNS diseases through the blood‒brain barrier remains to be further studied. Third, most NLRP3 inflammasome inhibitors are still in preclinical research, and the FDA has not approved any NLRP3-specific drugs for clinical use. Fourth, some of these drugs have other known targets in addition to NLPR3, so it is necessary to further determine whether there are off-target effects in the treatment of diseases in the future.

The clinical therapeutic effects of NLRP3 inflammatory body inhibitors on CNS diseases need to be further studied. Owing to drug selectivity, drug metabolism, and significant potential side effects, how can we optimize drug design? Can monoclonal antibodies and RNA interference techniques be applied? Are there new targeted drugs that are more effective? Is combination therapy with different mechanisms needed?

In conclusion, although the development of NLRP3 inhibitors faces challenges, their potential clinical applications are promising. Future studies are needed to clarify the role of NLRP3 in different types of CNS diseases and explore personalized treatment approaches. In addition, the development of clinical trials will provide important evidence for the safety and efficacy of NLRP3-targeted therapies.
